# Ubiquitination Process Mediates Prostate Cancer Development and Metastasis through Multiple Mechanisms

**DOI:** 10.1007/s12013-023-01156-x

**Published:** 2023-10-17

**Authors:** Wen Li, Zhiyu Wang

**Affiliations:** Department of Immuno-Oncology, The Fourth Hospital of Hebei Medical University, Shijiazhuang, China

**Keywords:** Prostate cancer, Metastasis, Ubiquitination, E3 ubiquitin ligase, PROTAC

## Abstract

Prostate cancer (PCa) is a common malignant tumor in men, when the disease progresses to the advanced stage, most patients will develop distant metastasis and develop into castration-resistant prostate cancer (CRPC), resulting in increased mortality. Ubiquitination is a widespread protein post-translational modification process in the biological world, and it plays an important role in the development and transfer of PCa. E3 ubiquitin ligase plays an important role in the specific selection and role of substrates in the process of ubiquitination ligase. This review will briefly introduce the ubiquitination process and E3 ubiquitin ligase, focus on the recently discovered multiple mechanisms by which ubiquitination affects PCa development and metastasis, and a summary of the current emerging proteolysis-targeting chimeras (PROTAC) in the treatment of PCa.

## Introduction

PCa is the most common malignant tumor in men, accounting for about 26% of all malignant tumors [1]. Among them, the incidence of metastatic prostate cancer in men aged 45 to 64 continues to increase [2], and the increased mortality of PCa is mainly due to the development of the disease to the late stage of metastasis [3]. For the early diagnosis of PCa, prostate-specific antigen has greatly improved its diagnostic efficiency, enabling patients with early-stage prostate cancer to achieve a five-year survival rate of about 100% [4]. However, the five-year survival rate of advanced PCa is low, only about 28%, one of the reasons is that multiple distant metastases, such as bone metastases and lymph node metastases, often occur in the advanced stage. Since the effects of androgen deprivation therapy (ADT) in metastatic PCa patients were first reported in 1941, inhibition of androgen receptor (AR) signaling by ADT has been the mainstay of treatment for metastatic PCa [5], although this therapy has brought about 1–2 years of remission, the emergence of metastatic castration-resistant prostate cancer (mCRPC) makes cancer cells resistant to drugs, which leads to poor efficacy. Therefore, it is very important to explore the therapeutic targets of metastatic PCa at present.

During biological development, the balance of protein synthesis and degradation affects various processes such as cell proliferation, differentiation, and apoptosis. Among them, ubiquitination is one of the important regulatory processes of protein post-translational modification, mainly composed of ubiquitin (Ub), ubiquitin-activating enzyme E1, ubiquitin-conjugating enzyme E2, and ubiquitin ligase E3. Dysregulation of the ubiquitin pathway is associated with many diseases including cancer, involved in PCa [6]. By labeling substrates with ubiquitin, E3 ubiquitin ligases provide these substrates with a new platform for protein-protein interactions, which in turn alter their activity, localization, and/or further interactions to elicit diverse biological signals [7]. Therefore, through the research and understanding of E3 ligase, it is very meaningful for the diagnosis and treatment of metastatic PCa.

This review will briefly describe the ubiquitination process and E3 ubiquitin ligase, it also focuses on the recently discovered ubiquitination through the effects of epithelial-mesenchymal transition (EMT), AR, cancer stem cells (CSCs), energy metabolism, cell cycle, and other mechanisms lead to the occurrence and transfer of PCa; in the last part of the article, we briefly summarize the current treatment methods for PCa by E3 ubiquitin ligase.

## Ubiquitination

### Ubiquitination Process

Among various post-translational modifications, protein ubiquitination is a common and important process in cells [8]. The ubiquitination process of the substrate is a cascade reaction process: first, the ubiquitin-activating enzyme E1 activates the Ub molecule and forms a thiol ester bond between the 76th glycine at the C-terminus of the Ub molecule and the cysteine of the E1 enzyme. This process requires the consumption of adenosine triphosphate (ATP) [9]. The Ub-E1 intermediate then transfers the activated Ub molecule to the ubiquitin-conjugating enzyme E2 [10]; finally, the ubiquitin ligase E3 specifically recognizes the substrate and catalyzes the E2 enzyme to transfer Ub to the substrate for post-translational modification of the substrate [11–13].

**Fig. 1:**
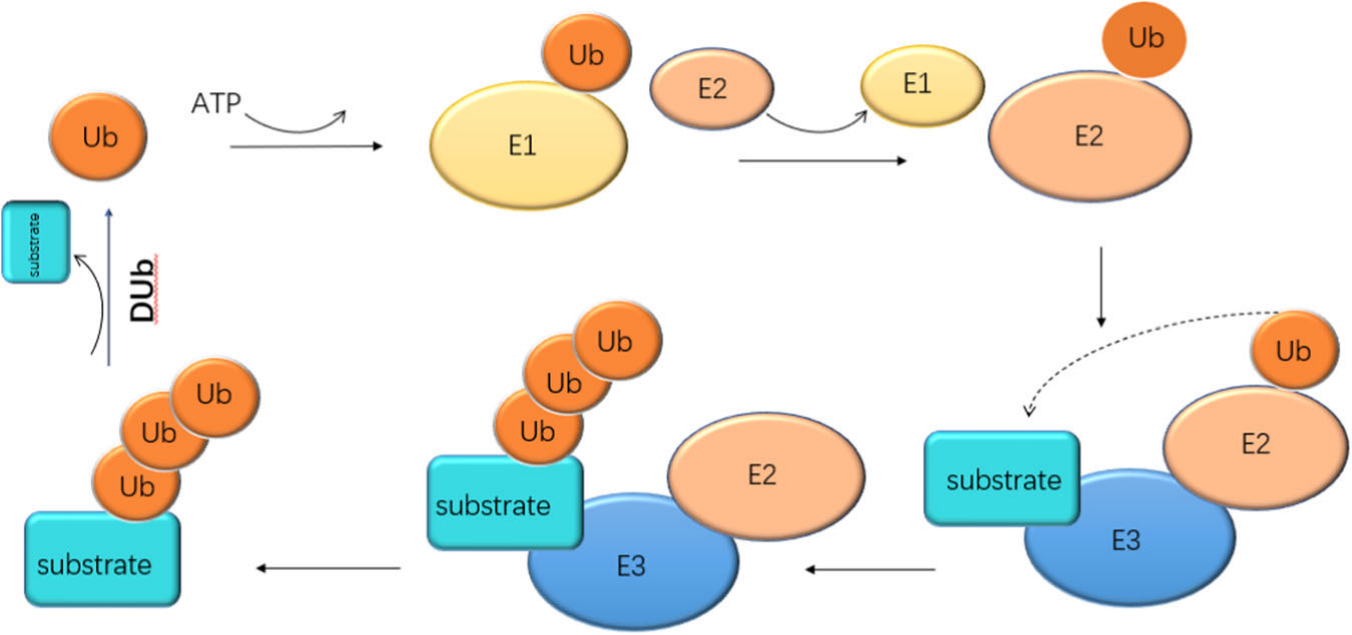
The process of ubiquitination. The ubiquitination process of the substrate is a cascade reaction process: first, E1 activates the Ub molecule, the Ub-E1 intermediate transfers the activated Ub molecule to E2, and finally, E3 specifically recognized the substrate and catalyzes E2 to transfer Ub to the substrate for post-translational modification of the substrate

Ubiquitination is a dynamic equilibrium process, which can be counterbalanced by about 99 deubiquitinases (DUb). The balance between ubiquitination and DUB is closely related to the regulation of protein-level activity, and DUB also maintains cellular ubiquitin levels by processing newly synthesized ubiquitin precursors and recycling ubiquitin from degraded proteins [14]. (Fig. 1).

### E3 Ubiquitin Ligase

At present, more than 600 E3 ubiquitin ligases have been identified in humans. According to different structures and functions, they can be divided into three types: RING-type, HECT type, and RING-IBR-RING (RBR) type [7, 15].

**Fig. 2:**
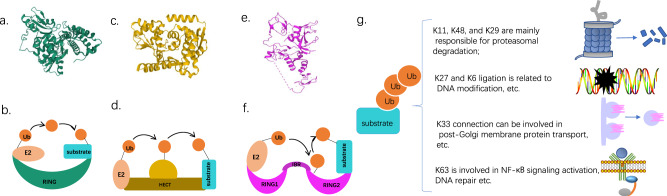
**a**, **b** RING E3-catalyzed ubiquitn transfer. **a** Structure of native c-Cbl (PDB 10.2210/pdb2Y1M/pdb). **b** The RING domine combines the E2-Ub and substrate protein, then directly transfers the Ub to the substrate. **c**, **d** HECT E3-catalyzed ubiquitin transfer. **c** Crystal structure of the HECT domain of human WWP2(PDB 10.2210/pdb4Y07/pdb). **d** The HECT domine first transfers the Ub molecule to the HECT domain, and then transfer the Ub molecule to the target protein. **e**, **f** RBR E3-catalyzed ubiquitin transfer. **e** Structure of Parkin E3 ligase (PDB 10.2210/pdb4l1H/pdb) (**f**). The RING1 domain can bind the Ub-loaded E2-binding enzyme, and the RING2 domain transfers the Ub molecule to the substrate for subsequent catalytic reactions. **g** Different biological effects can be produced, when the type of Ub connection is different

The specific ways in which the four ubiquitin ligases catalyze the transfer of Ub molecules from E2-conjugating enzymes to substrates are different. For example, the RING E3 ligases are characterized by their RING or U-box fold catalytic domain, which directly transfers the Ub molecule on the E2-binding enzyme to the target protein [16, 17]. (Fig. 2a, b) HECT-type E3 ligase has a conserved C-terminal catalytic domain, it first transfers the Ub molecule to the HECT domain and forms a thioester bond intermediate with the Ub molecule, and then transfer the Ub molecule to the target protein [18]. (Fig. 2c, d) The RBR-type E3 ligase has two RING structures, in which the RING1 domain can bind the Ub-loaded E2-binding enzyme, and the RING2 domain transfers the Ub molecule to the substrate for subsequent catalytic reactions [19]. (Fig. 2e, f).

Polyubiquitination begins with the attachment of a single ubiquitin (Ub) molecule to a lysine residue on a protein substrate. Different biological effects can be produced depending on the type of Ub connection. For example, the function of K48-linked chains is related to classic protein degradation [20]. K11 and K29 appear to have the same function [21, 22]. K11-linked chains are used for cell cycle regulation [23], and K27 and K6 are related to DNA damage response and other related functions [24, 25]. K33 can be involved in post-Golgi membrane protein transport [26], while K63 is involved in NF-κB signaling activation and DNA repair [27]. It should be noted that these chains can be either homotypic or heterotypic (Fig. 2g).

## Ubiquitination Mediates the Development and Metastasis of Prostate Cancer through Multiple Mechanisms

### EMT

EMT is a key event in tumor metastasis, allowing quiescent tumor cells to acquire mesenchymal features with high migratory rates and migratory potential [28]. In PCa, EMT is also a key process leading to metastases in the late stages of the disease, which is typically characterized by up-regulation of mesenchymal markers such as vimentin and N-cadherin and down-regulation of epithelial markers such as E-cadherin [29]. At the same time, many regulatory factors can lead to the EMT process, the following section expands to describe the process of ubiquitination and that various E3 ligases can promote or inhibit this process by regulating various EMT-related molecules, and plays a different role in the occurrence, development, and metastasis of PCa.

The activation of cSRC promotes a stable association between EMT and cell invasion transcription factors such as SNAIL, SLUG, and ETS1 [30]. Moro et al. [31] found that the E3 Ub ligase FBXL7 can recognize the SH3 domain of c-SRC upon phosphorylation at Ser104 and specifically target c-SRC for proteasomal degradation, to inhibit prostate metastasis. ILEI has been shown to regulate tumor progression and be upregulated at the translational level during EMT [32]. Sun et al. [33] showed, through co-immunoprecipitation and other experiments, that UBE4A targets ILEI and inhibits the migration and invasion of PCa cells in vitro, through ubiquitination and degradation. The correlation between PROX1 expression and cancer progression has been explored in several cancer types. In PCa, PROX1 overexpression enhances the accumulation of the HIF1α protein by inhibiting the Ub pathway, enhancing cell migration by inhibiting E-cadherin, upregulating vimentin and matrix metallopeptidase, and inducing EMT responses [34].

β-catenin is a key regulator of cancer-associated EMT [35]. GSK-3β can recognize and phosphorylate β-catenin, generating a site that β-TrCP E3 Ub ligase can bind to, triggering the ubiquitination and degradation of β-catenin [36], thereby eliminating its EMT-promoting effect and reducing the metastasis of tumor cells. Therefore, the GSK-3β/β-catenin axis plays a key role during EMT. Tian et al. [37] showed that melatonin inhibits nuclear translocation by enhancing the phosphorylation of cytoplasmic β-catenin and thus moderating its degradation through ubiquitination. Wang et al. [38] found that CARMIL3 is required for the migration and invasion of PC3 cells, and demonstrated that CARMIL3 can maintain Cdh1 transcription by inhibiting the transcriptional repressor ZEB2. This repressor interferes with β-catenin binding to the destruction complex, protecting β-catenin from being degraded via ubiquitination. Microtubule stability is known to be related to the regulation of EMT, and knockdown of human tubulin β-IVa (TUBB4A) reduces GSK3β ubiquitination and degradation, increasing the expression of E-cadherin in PCa cells, while the expression of N-cadherin and vimentin decrease [39]. Therefore, it has been found that, in the GSK-3β/β-catenin axis, the ubiquitination-proteasomal degradation system can inhibit EMT.

In cancer, TGF-β/SMAD is one of the most prominent inducers of EMT. TGF-β/SMAD binds to TGF-β ligands and receptors, where it phosphorylates and activates Smad2 and Smad3, which then bind to Smad4 to regulate gene transcription [40]. TIF1γ can inhibit TGF-β signaling by mediating the ubiquitination of Smad4 [41]. Qi et al. [29] and Lan et al. [42, 43] proved that, in PC-3 cells, TIF1γ overexpression correlated positively with the epithelial marker E-cadherin, but negatively with mesenchymal markers such as vimentin and N-cadherin. In addition to E3 ligases that are negatively correlated to EMT, many E3 ligases are positively related to the EMT process in PCa cells. ITCH is an E3 ligase, and its positive regulation of TGF-β signaling is dependent on the ubiquitination and subsequent degradation of Smad7. Therefore, ITCH may promote EMT in breast cancer cells, by downregulating Smad7 [44]. Under normal circumstances, the E3 Ub ligase adapter SPOP is involved in Ub-mediated ITCH degradation, but the F133L mutation in SPOP in PCa leads to ITCH-mediated EMT and promotes the development of the disease [45]. The N-myc downstream regulatory gene 1 (NDRG1) is a tumor suppressor gene in multiple cancers, including PCa. Its overexpression downregulates key signaling proteins and pathways, including those mediated by TGFβ and others [46]. Gamell et al. [47] demonstrated that E6AP may play a pro-metastatic role in PCa, by downregulating NDRG1, which in turn downregulates the TGF-β signaling protein that is affected by NDRG1, promoting EMT.

This is the classic Ub-proteasome degradation system in the process of ubiquitination, although E3 Ub ligase can also have other biological effects. TRAF6, for example, is an unconventional E3 ligase that activates key regulatory proteins via Lys-63-linked polyubiquitination [48]. Overexpression of TRAF6 in PCa upregulates the expression of the transcription factor SLUG, and thus enhances EMT. TRAF6 overexpression can also closely affect EMT, by mediating the NF-κB signaling pathway [49]. Singh et al. [50] performed a reversed-phase protein array study using cell lysates from PC3 cells that expressed two different shTRAF4 or control shRNA plasmids, and found numerous EMT and invasion-related proteins. By contrast, TrkA-knockout cells showed reduced expression of TRAF4 target genes. They thus confirmed that TRAF4 and TrkA interact to regulate cell invasion-related gene expression and cell invasion. They also concluded that TRAF4-mediated TrkA ubiquitination over-activated TrkA kinase activity on a lysine cluster (K523_44_47) close to the kinase domain and played a non-canonical proteasomal role in promoting EMT during the progression of PCa.

**Fig. 3:**
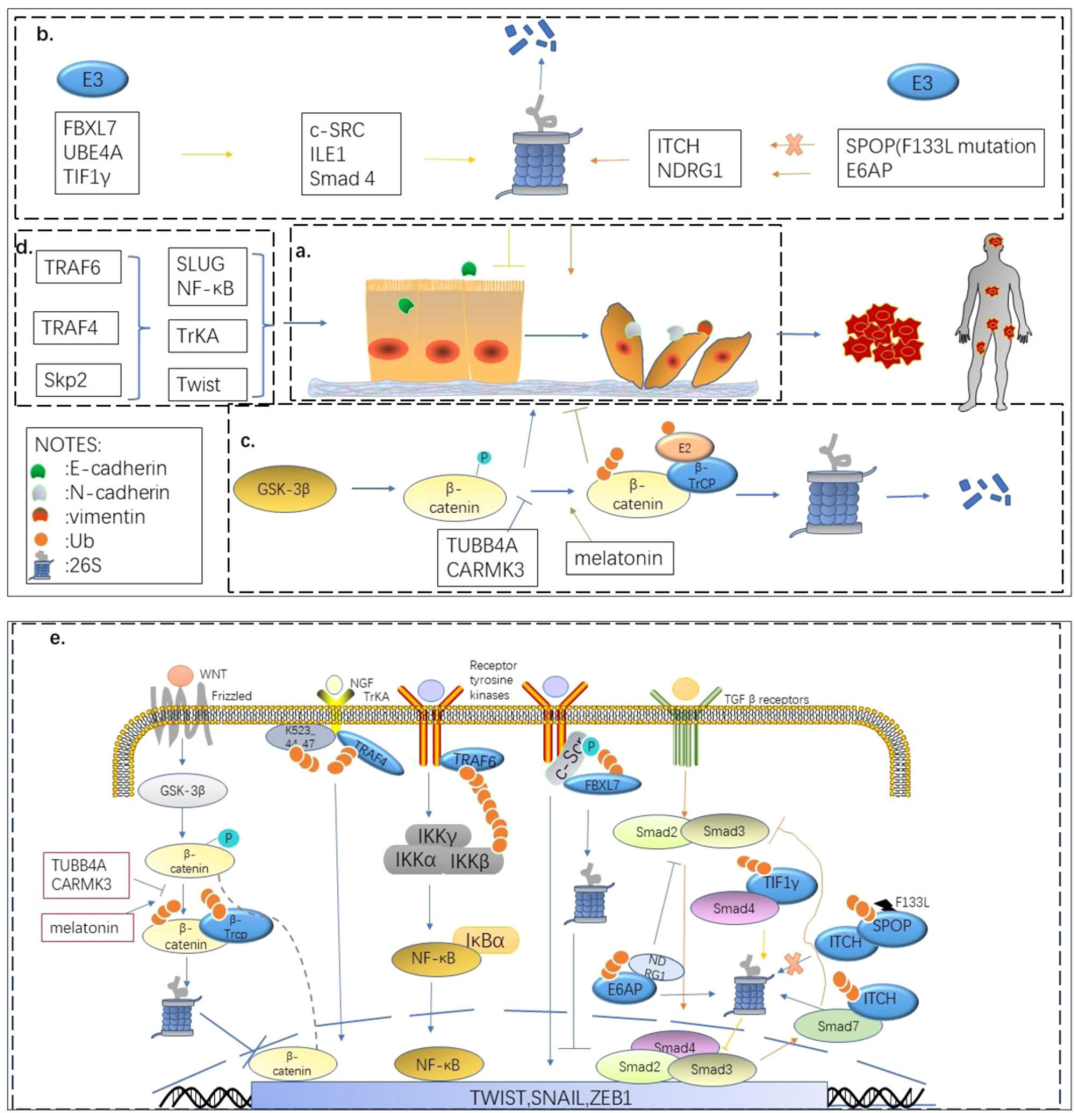
**a** The process of EMT. **b** Many E3 can through the 26s proteasome degradation system influence the EMT process. **c** GSK-3β/β-catenin axis can be inhibited through ubiquitination, at the same time, the process can be influenced by other molecular. **d** E3 can also play other biological effects in the EMT. **e** Part mechanism of E3s regulate the EMT

In addition to TRAF6, SKP2, a key component of the E3 Ub ligase SCF complex, also plays a role in non-26S proteasomal degradation, which in turn regulates EMT in PC. Twist, a transcription factor that exerts biological functions through transcriptional events in the nucleus, is a key driver of EMT in carcinogenesis, and is upregulated in 90% of malignant PCa tissues [51]. One of the mechanisms of its upregulation may be that Skp2 stabilizes and activates Twist through ubiquitination, by simultaneously antagonizing β-TrCP. TrCP can ubiquitinate the proteasome to degrade Twist and promote the ubiquitination of K63 but not K48-linked Twist, thereby promoting EMT [52]. Mickova et al. [53] also found a co-regulatory relationship between SKP2 and SLUG that promotes EMT, which could be inhibited by ubiquitination (Fig. 3).

### AR

AR is a key driver of PCa pathophysiology, regulates cancer cell proliferation, metabolism, and migration, is an empirical therapeutic target for PCa [54] and is crucial for the development of resistance to ADT [55]. Historically, inhibition of AR signaling by ADT has been the mainstay of metastatic PCa therapy for over 70 years, since Charles Huggins first reported the effects of ADT in patients with metastatic PCa in 1941 [5]. Although ADT is initially effective in approximately 90% of patients with PCa, the disease inevitably develops into fatal CRPC [56], which results in multiple distant metastases that seriously endanger patients’ lives. Various E3 Ub ligases regulate AR activity through ubiquitination, which, in turn, affects PC development and metastasis.

**Fig. 4:**
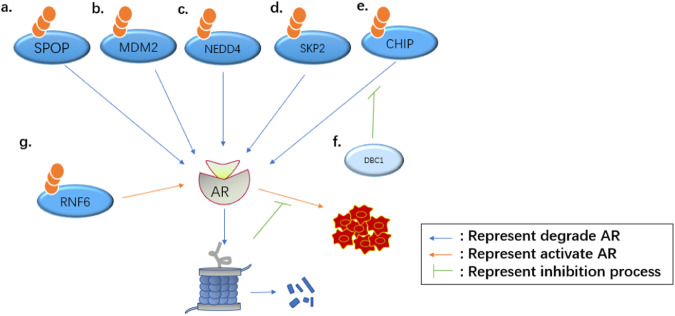
E3s can through different ways to regulate the AR signal in PCa, and ultimately have different effects. The regulate process can be regulated by other molecular. From the (**a**–**e**), E3s are SPOP, MDM2, NEDD4, SKP2, CHIP that can degrade AR, inhibit PCa progression. The (**f**) demonstrated that DBC1 competes with CHIP to bind AR-V7 to inhibit its mediated AR-V7 ubiquitination and degradation. The (**g**) demonstrated that RNF6E3 promotes AR transcriptional activity, promote PCa progression

SPOP not only modulates the EMT process, but also affects PCa progression by mediating AR degradation (Fig. 4a). An et al. [56] proved that AR is the substrate of SPOP through a number of experiments. In PCa cells, unmutated SPOP promotes the degradation of full-length wild-type AR (AR-WT), and acts as a tumor suppressor. Geng et al. [57] used biochemical techniques to find that, in androgen-dependent PCa cells and non-dependent cells, WT-SPOP can directly bind to the SBC motif in the AR hinge region, to promote its ubiquitination and degradation. However, this binding activity can be abolished by SPOP mutations in PCa, resulting in AR stabilization and the promotion of cancer cell proliferation and metastasis.

In addition to SPOP, other E3 Ub ligases can also degrade AR through ubiquitination. In PCa cells, MDM2 can ubiquitinate and degrade AR, mainly through the PI3K/Akt phosphorylation pathway. When activated, Akt phosphorylates AR and MDM2, increasing their binding and leading to the proteasomal degradation of AR (Fig. 4b) [58]. Skp2-mediated AR degradation, by contrast, occurs in an Akt/mTOR-independent manner [59]. It has also been shown that, in PCa cells, the ubiquitination and degradation of AR by MDM2 can lead to AR(-) CSCs, which ultimately promote the occurrence of metastatic tumors [60]. AR can also be degraded by a number of other E3 Ub ligases such as CHIP, NEDD4, and SKP2 (Fig. 4e–g) [60].

In addition to acting on morphologically normal AR, there is also a mechanism against AR mutant degradation in PCa. For example, DBC1 is a co-activator of various transcription factors that can directly enhance the DNA-binding activity of AR-V7. It can also inhibit the CHIP-mediated ubiquitination and degradation of AR-V7, by competing with it and thereby stabilizing and activating it. This increases CRPC and promotes cancer metastasis (Fig. 4f) [61].

E3 Ub ligase can also regulate AR activity in other ways. RNF6 E3 Ub ligase is upregulated in PCa, and can induce polyubiquitination at K845 of AR. The polyubiquitin chain in the AF2 domain of AR can in turn recruit a host of coregulators that promote the transcriptional activity of AR (Fig. 4g) [62]. Most PROTAC small-molecule drugs developed for PCa, such as ARV-110 and ARCC-4, mainly target AR [63].

### CSCs

Most cancers contain a small number of cells with stem-like properties called CSCs, which function promote tumor initiation and self-renewal [64]. The concept of CSCs provides a new perspective for understanding the occurrence, development, metastasis, and prognosis of tumors, as well as tumor resistance to therapy. CSCs exhibit multidirectional differentiation, self-renewal, and robust tumorigenesis [65]. In PCa, PCa stem cells are considered tumor initiation and maintenance cells [66]. E3 Ub ligases such as CUL4B, SPOP, Skp2, and MDM2 can inhibit, promote, and maintain PCa stem cells through various mechanisms, thereby affecting the progression and metastasis of the malignancy.

**Fig. 5:**
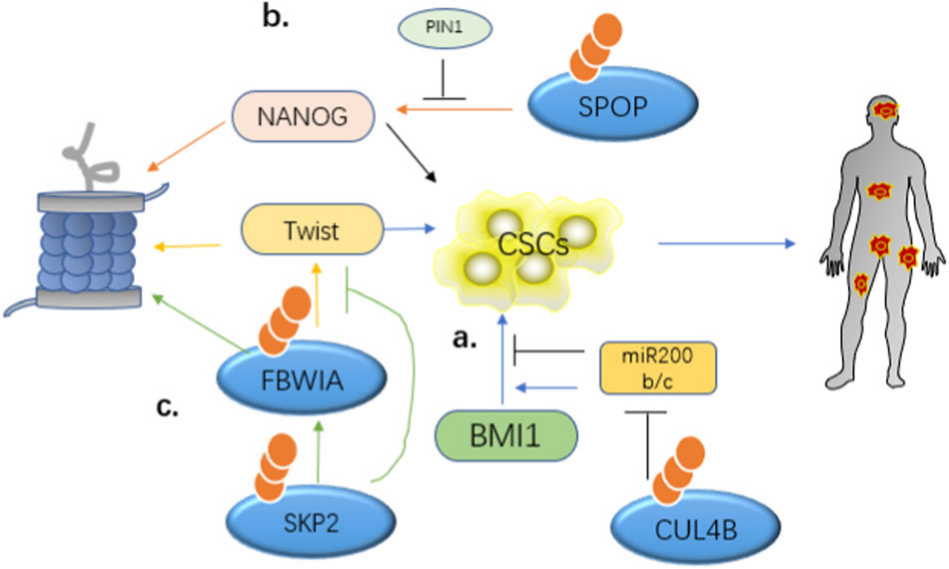
E3s influence PCa through regulate CSCs. **a** CUL4BE3 inhibits the transcription of BMl1 by causing trimethylation of miR200b/c, promoting BMl1-regulated PCa stem cell characteristics. **b** SPOP causes NANOG degradation, PIN1 inhibits polyubiquitination and degradation of SOPO. **c** Skp2 degrades FBW1A and inhibits its degradation of Twist, ultimately promoting tumor development and metastasis

BMI1 is a major component of polycomb repressive complex 1 (PRC1), which contributes to the maintenance of CSCs in various malignancies. In PCa, the function of CUL4B E3 Ub ligase, through its transcriptional repressive activity, leads to the trimethylation of miR200b/c, in turn inhibiting its transcription and promoting BMI1-regulated PCa stem cell traits that lead to PCa progression and metastasis (Fig. 5a) [67].

Embryonic CSCs express pluripotency-related transcription factors such as NANOG. The expression of these transcription factors is closely associated with the development of cancer [68]. Wang et al. [69] reported that SPOP can interact directly with the S68 residue in the SBC group of NANOG in PCa cells, leading to the proteasomal degradation of NANOG, which then inhibits the self-renewal and stem cell-like characteristics of PCa. Zhang et al. [70] also proved that SPOP degrades NANOG through the proteasomal system, thereby reducing stem cell characteristics in embryonic stem cells and PCa. It has also been verified that the upstream factor of NANOG, Pin1, stabilizes SPOP, inhibits its polyubiquitination and degradation, and promotes CSC characteristics that are beneficial to tumorigenesis (Fig. 5b).

As previously described, the transcription factor Twist is a key driver of CSC characteristics in cancer cells. Ruan et al. [52] proved that, in PCa cells, Skp2 can degrade FBW1A through the proteasomal pathway and inhibit the degradation of Twist. This eventually stabilizes its expression and enhances CSC self-renewal in PCa, promoting tumor development and metastasis (Fig. 5c). There is also an AR(-) phenotype CSC population in PCa, wherein MDM2 promotes AR degradation, which is essential for maintaining PCa stem cell homeostasis and promoting CRPC [60].

### Energy Metabolism

Tumor cells generally reprogram their metabolism [71], to generate cellular plasticity, facilitate the adaptation of cancer cells to unfavorable microenvironments, and promote disease progression [72]. Around 100 years ago, Otto Warburg discovered that some cancer cells can convert glucose into lactate through aerobic exercise, whereas most normal cells in the body use glucose for oxidative metabolism. This property is known as the “Warburg” effect [73]. High levels of aerobic glycolysis not only meet the energy needs of cancer cells, but also promote the synthesis of macromolecules for rapid tumor proliferation and metastasis [74]. Mitochondria also play an important role in tumor metabolism [75], contributing to tumor invasion and metastatic dissemination through redox balance and mitochondrial dynamics. Parkin and VHL E3 Ub ligase, the UBE2O E2 conjugating enzyme with E3 activity, and the ubiquitination process, affect PCa progression by mediating energy metabolism.

Agarwal et al. [76] demonstrated that Parkin suppresses tumor growth by targeting glycolysis and mitochondrial networks. The Parkin ligase induces the degradation of the non-oxidative PPP enzyme TK, leading to glycolytic starvation, loss of ATP production, and generation of mitochondrial ROS in tumor cells. The mitochondrial kinetic effectors MFN2 and RHOT1, and the non-oxidative phase (TKT) of PPP can be degraded, ultimately leading to acute metabolic and oxidative stress and the inhibition of subcellular mitochondrial transport. This prevents primary and metastatic tumor growth in vivo. VHL can target HIF-1α for proteasomal degradation under conditions of normoxia. However, Fan et al. [74] found that PLCε inhibited this proteasomal degradation in a pVHL-dependent manner in PCa cells, regulated the stability of HIF-1α, and thus promoted PCa development and metastasis.

Although UBE2O is an E2 ligase, it also possesses E3 ligase properties. Vila et al. [77] showed that the deletion of one or both alleles of UBE2O resulted in delayed tumorigenesis, as well as reduced tumor growth and metastasis rates, in a mouse model for PCa. Its specific tumor-promoting and pro-metastatic mechanisms involve the ubiquitination and degradation of AMPKα2. This promotes the activation of the mTOR-HIF1α pathway, leading to its upregulation, which is closely related to growth-promoting, glycolytic, and biosynthesis pathways.

### Cell Cycle

Correct progression of the cell cycle depends on an ordered sequence of events that include DNA replication, chromosome condensation, and cytokinesis [78]. One of the important characteristics of tumor cells is their cell cycle and energy metabolism [79]. The occurrence and development of various cancers are characterized by a highly activated cell cycle, the ability of cancer cells to replicate indefinitely, and tissue invasion/metastasis, which are among the main causes of death in patients with cancer. Ubiquitination and the SPOP E3 Ub ligase play important roles in tumor development, by regulating the cell cycle.

Cell division cycle-associated protein 5 (CDCA5) regulates sister chromatid aggregation and segregation to ensure accurate chromosome distribution in mitotic and meiotic cells [80]. CDCA5 deletion results in mitotic arrest and the complete loss of sister chromatid cohesion [81]. Luo et al. [82] found that SPOP could regulate the ubiquitination and degradation of the tumor-promoting protein CDCA5 through the AKT pathway in DU145 cells. This inhibited, either partially or fully, the expression of the CDCA5 protein, thereby inhibiting tumor cell proliferation and promoting apoptosis. Cyclin E1 is an important protein involved in cell cycle progression. Improper degradation of cyclin E1 on chromatin can lead to abnormal DNA replication and increase the risk of gene mutations and tumorigenesis [83]. Ju et al. [84] found that in PCa cells, SPOP directly interacts with cyclin E1 through the MATH domain to promote its ubiquitination and degradation, which in turn inhibits the cancer-related phenotypes induced by cyclin E1 expression.

TUBB4A is a member of the β-tubulin family. In most normal tissues, there is little or no TUBB4A expression. Gao et al. [39] showed, through a functional analysis, that TUBB4A/GSK3β binds to the N-terminus of MYH9 in PCa cells. TUBB4A-KO reduces the MYH9-mediated ubiquitination and degradation of GSK3β, which in turn inhibits cyclin D1, acting to reduce spontaneous tumor growth and metastasis.

Polo-like kinase 1 (PLK1) plays an important role in regulating cell cycle processes such as centrosome maturation, spindle assembly, mitotic exit, and cytokinesis. Many of its functions oppose those of Smad4. Gao et al. [85] showed that PLK1 and PELO can directly bind to different domains of Smad4 to form a protein complex. PLK1 promotes the ubiquitination and degradation of Smad4 by phosphorylating it at Thr197 in PCa cells, using PELO as a coenzyme. The biological effects of PLK1 inhibitors on cell growth and metastasis can be restored by knocking out Smad4, which in turn promotes tumor development and metastasis by affecting the cell cycle.

### Other

The process of ubiquitination through an E3 Ub ligase can also affect the development and metastasis of PCa through other mechanisms as well, such as the regulation of cancer cell dormancy and endoplasmic reticulum stress.

In a subset of PCa patients, bone metastases can occur after incubation periods of years or even decades. One key mechanism that underlies this phenomenon is that Wnt5a from the osteoblast niche activates non-canonical ROR2/SIAH2 signaling to ubiquitinate β-catenin via SIAH2, inhibiting standard Wnt/β-catenin signaling and thereby reducing invasion and bone metastasis [86]. One in vitro analysis showed that FBXL4 plays a role in regulating cell migration and invasion, and can prevent cancer metastasis to bones by downregulating ERLEC1 [87]. ERLEC1 is a molecular chaperone that plays a role in the endoplasmic reticulum stress response, and is related to cancer invasion and metastasis [88].

## Therapy

The importance of the ubiquitination process and E3 Ub ligases in cancer development and metastasis is self-evident, and a number of studies have shown that dysregulation of the ubiquitination process is closely related to cancer (including PCa). Therefore, E3 Ub ligases represent a promising therapeutic target for PCa [89, 90], and have received much attention in this regard in recent years. However, current research on E3 Ub ligase inhibitors for PCa has mainly focused on putative targets, and there is a lack of definitive clinical trials and applications.

**Fig. 6:**
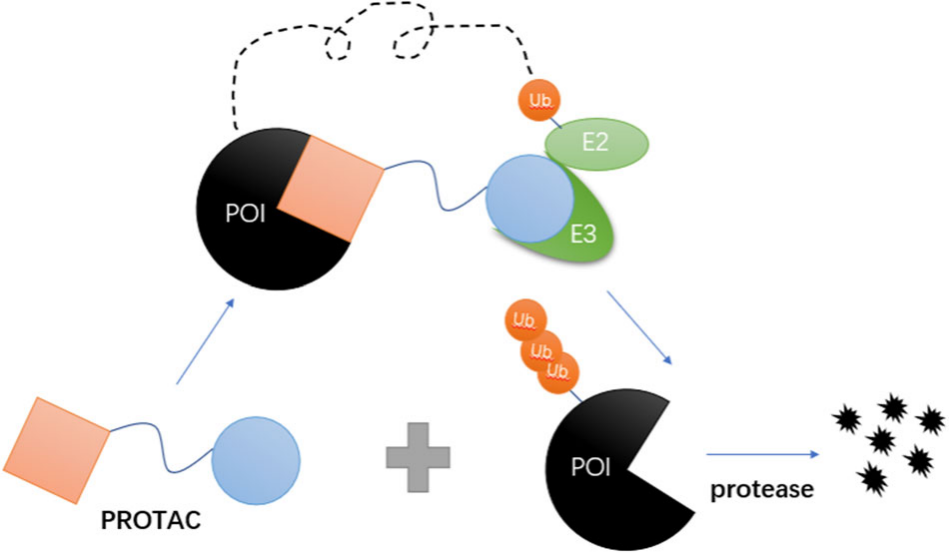
The process of PROTAC-mediated ubiquitination and proteasomal degradation of POI. PROTAC consists of ligands of POI and E3 ubiquitin ligase, which can bind both E3 ubiquitin ligase and target protein, and is degraded by endogenous 26S proteasomes

Newly emerging drugs that have shown some promise in this regard have targeted downstream protein degradation through the PROTAC approach. The concept of PROTAC, proposed by Crews and Deshaies in 2001 [91], represents a new way of targeting and degrading specific proteins. PROTAC is a bifunctional hybrid molecule consisting of a protein of interest (POI) ligand and an E3 Ub ligase ligand. The two are covalently connected, primarily by a linker composed of 5–15 carbon or other atoms. It can therefore bind both an E3 Ub ligase and a target protein at the same time, recruit the ligase after binding to the POI, and drive the ubiquitination of the target protein so it is degraded by the endogenous 26S proteasome [92, 93]. Importantly, PROTACs can also be recycled, making them more efficient at degrading large numbers of proteins [92]. Fig. 6 illustrates the PROTAC principle visually. Although there are hundreds of known E3 Ub ligases, only a a small fraction is used for PROTAC. These include Von Hippel-Lindau (VHL), cereblon (CRBN), inhibitor of apoptosis proteins (IAPs), and MDM2, with VHL and CRBN being the most widely used [94].

For many years, one of the main treatment methods for PCa has been ADT. However, most patients treated by ADT relapse, with cancers that metastasize and eventually progress to mCRPC with a much higher mortality rate. AR-dependent proliferation is one of the main mechanisms behind this phenomenon. Therefore, there have been many recent reports of drugs that specifically target AR using PROTAC. ARV-110, discovered by scientists at Arvinas Corporation, was the first potent orally-active AR degrader to enter clinical development that showed promising clinical activity and safety [95]. ARV110 is currently in a phase II clinical trial (NCT03888612), and researchers are actively exploring whether its dosing can be increased [96]. In various prostate cancer cell lines, ARV-110 degrades 95–98% of AR. In wild-type AR models, ARV-110 has shown similar efficacy to benzalutamide at lower doses. In drug-resistant models, ARV-110 reduced tumor growth by 70–100%. However, the clinical application of this drug has not yet been extensively explored, and there is a lack of definitive detection, statistical data, and countermeasures related to adverse reactions from its use.

ARCC-4 is another recently developed PROTAC, designed by Salami et al., that targets AR [97]. ARCC-4 is a low nanomolar AR degrader that is capable of degrading ~95% of cellular ARs. In several different cellular models of PCa resistance, the degradation efficiency of the targeted AR protein was approximately 10× that of enzalutamide. At the time of writing this review, ARCC-4 has been further optimized for ARCC-4 to ARV-110, and has entered phase I clinical trials (NCT03888612) [98].

ARD-266, first reported by Han et al. [99] has also shown significant inhibitory effects on AR. ARD-266 effectively induces AR degradation in AR-positive (AR+) LNCaP, VCaP, and 22Rv1 PCa cell lines. ARD-266 reduced AR protein levels in AR+ PCa cell lines by more than 95% and effectively reduced the inhibition of AR-regulated gene expression. This PROTAC reduced AR protein levels in LNCaP and VCaP cells by >95%. However, this study did not explore the therapy in live animals.

In a study by Kregel et al. [100] the role of ARD-61 was demonstrated in a benzalutamide resistance model. Several models of isogenic prostate cell lines with high AR-V7 expression were established; however, they failed to affect the cytostatic effect of AR degraders, suggesting that AR-V7 is not a functional resistance mechanism to AR inhibition. These data provide compelling evidence that full-length AR remains an important carcinogenic driver of PCa that has developed resistance to AR antagonists, and highlights the clinical potential of AR degradants for the treatment of CRPC.

Chen et al. [101] reported that a highly efficient PROTAC-based AR degrader, compound A031, induced AR protein degradation in VCaP cell lines in a time-dependent manner. A031 is five times less toxic than enzalutamide, and has clinically suitable half-life (T_1/2_) and clearance (Cl) times. It was shown to significantly inhibit the growth of transplanted tumors in VCaP zebrafish by inducing AR degradation in a time-dependent manner. In VCaP cell lines, AR target proteins were almost completely degraded after treatment with 2 mM of A031 for 4.5 h. At a concentration of 8.3 mM, it inhibited tumor growth by 55% in VCap-transplanted zebrafish. Therefore, A031 provides further insights into the development of new drugs for PCa. This drug can hopefully be further validated using resistance-related models.

Lee et al. [98] developed MTX-23, which binds both the DNA-binding domain of AR and the VHL E3 Ub ligase. They identified 12 human PCa cell lines that were resistant to four FDA-approved SAT drugs: abiraterone, benzalutamide, apalutamide, and darutamide. When drug-resistant cells were treated with MTX-23, a reduction in cell proliferation and tumor growth was observed both in vitro and in vivo. These results suggest that MTX-23 is a novel small PROTAC molecule. Researchers have also begun exploring various modifications to MTX-23, designed to enhance its potency. Future research will likely focus on how MTX-23 interacts with AR-V7 and AR-FL as well as on establishing the necessary pharmacokinetics in preparation for anti-SAT CRPC clinical trials in men.

Han et al. [102] found that the inhibitory effect of ARD-69 on AR-positive PCa cell growth was 100× higher than that of AR antagonists. ARD-69 can effectively inhibit the growth of AR-positive LNCaP and VCaP AR+ PCa cell lines and completely degrade AR in these two lines at a concentration of <1 nM for 24 h. It effectively inhibited cell growth in LNCaP, VCaP, and 22Rv1 PCa cell lines, and was more than 100× more potent than the AR antagonists benzalutamide and apalutamide. A single dose of ARD-69 effectively reduced AR protein levels in tumor tissues that had been transplanted into mice. Further optimization of ARD-69 may eventually lead to new therapies for AR+, castration-resistant PCa. Kregel et al. [100] developed several AR-V7-high isogenic PCa models and used PROTAC-ARD-69 to demonstrate that AR-V7 is not a functional resistance mechanism for AR inhibition.

Xiang et al. [103] found that ARD-2585 could act as a highly orally-active PROTAC-based degrader of ARs. In VCaP cell lines with wild-type AR, and in LNCaP cell lines carrying T878A mutant AR mutants, ARD-2585 received a picomolar DC50 value and a > 98% Dmax. It also reduced AR protein levels by >80% at 0.1 nM in 22Rv1 cell lines that carried AR-V7 variants, and by >80% at 1 nM in MDA-PCA-2b cell lines that carried double AR mutations. ARD-2585 is very stable in liver microsomes and plasma, even without HERG inhibition. It shows good PK parameters in both intravenous and oral routes of administration in mice, and achieves a wide tissue distribution. Oral administration of ARD-2585 effectively reduced AR protein expression and inhibited growth in VCaP tumor tissues that had been transplanted into mice. It has the potential to be widely used in the treatment of AR+, PCa, as well as other human diseases in which AR plays a key role.

Han et al. [104] also found that ARD-2128 had good oral pharmacokinetics in mice. Mechanistic studies showed that ARD-2128 is a true PROTAC-based degrader of AR that strongly inhibits AR + AR-regulated genes in PCa cell lines in a dose- and time-dependent manner. ARD-2128 also effectively inhibited the growth of an AR-amplified VCaP PCa cell line and an AR-mutated LNCaP cell line. The oral bioavailability of ARD-2128 in mice reached 67% as it effectively reduced the expression of AR in tumor tissues and inhibited the expression of AR regulatory genes, thereby effectively inhibiting the growth of the tumors in the mice, without any signs of toxicity. That study supported the development of orally active PROTAC-based AR degraders for PCa treatment, and provided insights and guidance for the design and development of the drugs.

**Fig. 7:**
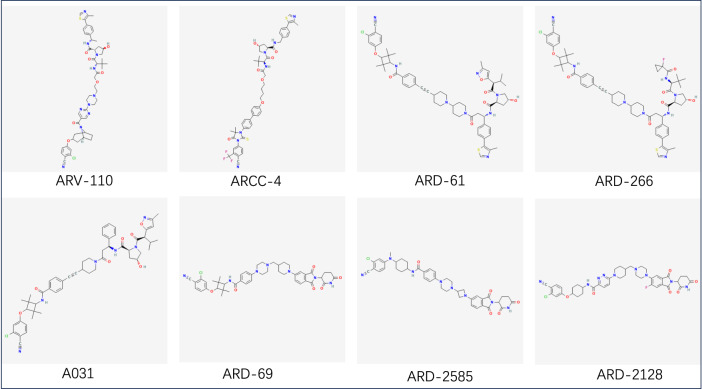
The structure of PROTAC. In order, is ARV-110, ARCC-4, ARD-61, ARD-266, A031, ARD-69, ARD-2585, ARD-2128. Download from PubChem (nih.gov)

The chemical structural formula of PROTAC is shown in Fig. 7, and the details are listed in Table 1.

**Table 1:** Some details about the PROTAC

Targeted drugs related to E3 Ub ligases have less impact on normal cell function and can achieve better therapeutic effects while minimizing side-effects. However, there are few specific POIs, E3 ligase targets, or clinical drug candidates in this field. It therefore remains to be seen whether PROTACs become effective drugs for the clinical treatment of PCa.

## Conclusion

PCa is a malignant epithelial tumor of the prostate with a high incidence in men. Distant metastasis often occurs when the disease progresses to an advanced stage, leading to increased mortality. Conventional surgery and chemotherapy can only improve the quality of life in many patients, without being able to significantly improve survival rates. Although ADT is initially effective in approximately 90% of PCa patients, the disease often eventually progresses to fatal CRPC. Because most patients have distant metastases at the time of diagnosis, precise targeted therapy is very important in order to control the disease, improve quality of life, and prolong survival time.

Ubiquitination is a common process in biology. It can mediate apoptosis, proliferation, differentiation, transcriptional endocytosis, DNA damage, and repair, through E1 Ub-activating enzyme, E2 Ub-conjugating enzyme, and E3 Ub ligase. Therefore, the relationship between ubiquitination and the development and treatment of PCa has received considerable attention.

Since E3 Ub ligases determine substrate specificity during ubiquitination, this article focuses on the proteasomal degradation, linkage, and activation of various E3 Ub ligases, by targeting substrate proteins. These functions affect EMT, AR, CSCs, energy metabolism, the cell cycle, and other pathways in the development of PCa, to either promote or inhibit its development and metastasis.

However, many accurate and specific E3 Ub ligases have not been identified, and only about 600 E3 Ub ligases have been discovered so far. Many E3 ligases can effectively regulate the progression of PCa, as well as other cancers; therefore, further exploration and verification of these drugs is highly warranted. In addition to the ubiquitination process, other protein post-translational modification processes can also affect PCa, such as ubiquitination [53, 105, 106], de-ubiquitination [107–110], methylation [31, 67, 72, 107, 109], and acetylation [111]. Therefore, it is beneficial to understand the occurrence, development, and metastasis mechanisms of PCa in further detail, by elucidating the process of protein post-translational modification in order to achieve effective prevention and treatment.

As the importance of ubiquitination’s role in cancer is self-evident, drugs targeting the E3 Ub ligase that affects PCa are also emerging. However, most of the research in this field is concentrated at the preclinical stage, and the translation of these research results into clinical applications is an urgent challenge that remains to be addressed. The emerging PROTAC technology is a new development that targets and eliminates substrate proteins through E3 Ub ligases. The current challenges surrounding the PROTAC system mainly include the lack of further animal model validation and clinical experiments, limited application of suitable E3 Ub ligases, and the need for further structural design. Most of the currently designed PROTAC delivery methods are oral, so other delivery methods can be used to verify their pharmacokinetics and distribution concentrations in vivo, as more effective delivery methods begin to be explored. As a small-molecule drug, PROTAC must be concentrated in sufficiently high levels to occupy the active sites of its targets, and must have a long enough half-life to continuously inhibit its target protein. Long-term, high-dose drug exposure not only increases the risk of adverse effects but also leads to cumulative toxicity. In clinical applications, attention should be paid to detecting adverse reactions to drugs, grading them, and improving relevant counter-measures. In addition, there remains much room for improvement with regard to the optimization of PROTAC doses. With a more comprehensive understanding of the physiological functions and recognition substrates of the >600 E3 Ub ligases that have been discovered thus far, applications in cancer therapy should become apparent and worthwhile. Although there has been considerable investment in research and experience in this field, there is still much uncertainty regarding whether this therapy type can actually be used in clinical practice.

## References

[1] Siegel RL, Miller KD, Fuchs HE (2021). Cancer Statistics, 2021. CA: A Cancer Journal of Clinicians.

[2] Dalela D, Sun M, Diaz M (2019). Contemporary trends in the incidence of metastatic prostate cancer among US men: Results from nationwide analyses. European Urology Focus.

[3] Ketchandji M, Kuo YF, Shahinian VB (2009). Cause of death in older men after the diagnosis of prostate cancer. Journal of the American Geriatrics Society.

[4] Kollmeier MA, Zelefsky MJ (2012). How to select the optimal therapy for early-stage prostate cancer. Critical Reviews in Oncology/Hematology.

[5] Komura K, Sweeney CJ, Inamoto T (2018). Current treatment strategies for advanced prostate cancer. International Journal of Urology.

[6] Shi D, Grossman SR (2010). Ubiquitin becomes ubiquitous in cancer: emerging roles of ubiquitin ligases and deubiquitinases in tumorigenesis and as therapeutic targets. Cancer Biology and Therapy.

[7] Buetow L, Huang DT (2016). Structural insights into the catalysis and regulation of E3 ubiquitin ligases. Nature Reviews Molecular Cell Biology.

[8] Antao AM, Tyagi A, Kim KS (2020). Advances in deubiquitinating enzyme inhibition and applications in cancer therapeutics. Cancers (Basel).

[9] Schulman BA, Harper JW (2009). Ubiquitin-like protein activation by E1 enzymes: The apex for downstream signalling pathways. Nature Reviews Molecular Cell Biology.

[10] Ye Y, Rape M (2009). Building ubiquitin chains: E2 enzymes at work. Nature Reviews Molecular Cell Biology.

[11] Deshaies RJ, Joazeiro CA (2009). RING domain E3 ubiquitin ligases. Annual Review of Biochemistry.

[12] Rotin D, Kumar S (2009). Physiological functions of the HECT family of ubiquitin ligases. Nature Reviews Molecular Cell Biology.

[13] Smit JJ, Sixma TK (2014). RBR E3-ligases at work. EMBO Reports.

[14] Clague MJ, Urbe S, Komander D (2019). Breaking the chains: Deubiquitylating enzyme specificity begets function. Nature Reviews Molecular Cell Biology.

[15] Zheng N, Shabek N (2017). Ubiquitin ligases: Structure, function, and regulation. Annual Review of Biochemistry.

[16] Fouad S, Wells OS, Hill MA (2019). Cullin Ring Ubiquitin Ligases (CRLs) in cancer: Responses to Ionizing Radiation (IR) Treatment. Frontiers in Physiology.

[17] Hatakeyama S, Nakayama K-II (2003). U-box proteins as a new family of ubiquitin ligases. Biochemical and Biophysical Research Communications.

[18] Weber J, Polo S, Maspero E (2019). HECT E3 ligases: A tale with multiple facets. Frontiers in Physiology.

[19] Spratt DE, Walden H, Shaw GS (2014). RBR E3 ubiquitin ligases: New structures, new insights, new questions. Biochemical Journal.

[20] Xu P, Duong DM, Seyfried NT (2009). Quantitative proteomics reveals the function of unconventional ubiquitin chains in proteasomal degradation. Cell.

[21] Zhang ZD, Li HX, Gan H (2022). RNF115 Inhibits the Post-ER Trafficking of TLRs and TLRs-Mediated Immune Responses by Catalyzing K11-Linked Ubiquitination of RAB1A and RAB13. Advanced Science.

[22] Gao P, Ma X, Yuan M (2021). E3 ligase Nedd4l promotes antiviral innate immunity by catalyzing K29-linked cysteine ubiquitination of TRAF3. Nature Communications.

[23] Bremm A, Komander D (2011). Emerging roles for Lys11-linked polyubiquitin in cellular regulation. Trends in Biochemical Sciences.

[24] Gatti M, Pinato S, Maiolica A (2015). RNF168 promotes noncanonical K27 ubiquitination to signal DNA damage. Cell Reports.

[25] Wu-Baer F, Lagrazon K, Yuan W (2003). The BRCA1/BARD1 heterodimer assembles polyubiquitin chains through an unconventional linkage involving lysine residue K6 of ubiquitin. Journal of Biological Chemistry.

[26] Yuan WC, Lee YR, Lin SY (2014). K33-linked polyubiquitination of coronin 7 by Cul3-KLHL20 Ubiquitin E3 ligase regulates protein trafficking. Molecular Cell.

[27] Ohtake F, Saeki Y, Ishido S (2016). The K48-K63 branched ubiquitin chain regulates NF-κB signaling. Molecular Cell.

[28] Chen T, You Y, Jiang H (2017). Epithelial-mesenchymal transition (EMT): A biological process in the development, stem cell differentiation, and tumorigenesis. Journal of Cellular Physiology.

[29] Chaffer CL, San Juan BP, Lim E (2016). EMT, cell plasticity and metastasis. Cancer and Metastasis Review.

[30] Guarino M (2010). Src signaling in cancer invasion. Journal of Cellular Physiology.

[31] Moro L, Simoneschi D, Kurz E (2020). Epigenetic silencing of the ubiquitin ligase subunit FBXL7 impairs c-SRC degradation and promotes epithelial-to-mesenchymal transition and metastasis. Nature Cell Biology.

[32] Chaudhury A, Hussey GS, Ray PS (2010). TGF-beta-mediated phosphorylation of hnRNP E1 induces EMT via transcript-selective translational induction of Dab2 and ILEI. Nature Cell Biology.

[33] Sun Y, Jia X, Gao Q (2017). The ubiquitin ligase UBE4A inhibits prostate cancer progression by targeting interleukin-like EMT inducer (ILEI). IUBMB Life.

[34] Wang B, Huang J, Zhou J (2016). DAB2IP regulates EMT and metastasis of prostate cancer through targeting PROX1 transcription and destabilizing HIF1alpha protein. Cellular Signalling.

[35] Li K, Zhang J, Tian Y (2020). The Wnt/beta-catenin/VASP positive feedback loop drives cell proliferation and migration in breast cancer. Oncogene.

[36] Stamos JL, Weis WI (2013). The beta-catenin destruction complex. Cold Spring Harbor Perspectives in Biology.

[37] Tian QX, Zhang ZH, Ye QL (2021). Melatonin inhibits migration and invasion in LPS-stimulated and -unstimulated prostate cancer cells through blocking multiple EMT-relative pathways. Journal of Inflammation Research.

[38] Wang H, Wang C, Peng G (2020). Capping protein regulator and myosin 1 Linker 3 Is required for tumor metastasis. Molecular Cancer Research.

[39] Gao S, Wang S, Zhao Z (2022). TUBB4A interacts with MYH9 to protect the nucleus during cell migration and promotes prostate cancer via GSK3beta/beta-catenin signalling. Nature Communications.

[40] Matsuzaki K (2011). Smad phosphoisoform signaling specificity: the right place at the right time. Carcinogenesis.

[41] Yu C, Ding Z, Liang H (2019). The roles of TIF1gamma in Cancer. Frontiers in Oncology.

[42] Qi G, Lu G, Yu J (2019). Up-regulation of TIF1γ by valproic acid inhibits the epithelial mesenchymal transition in prostate carcinoma through TGF-β/Smad signaling pathway. European Journal of Pharmacology.

[43] Lan X, Lu G, Yuan C (2016). Valproic acid (VPA) inhibits the epithelial-mesenchymal transition in prostate carcinoma via the dual suppression of SMAD4. Journal of Cancer Research and Clinical Oncology.

[44] Park SH, Jung EH, Kim GY (2015). Itch E3 ubiquitin ligase positively regulates TGF-beta signaling to EMT via Smad7 ubiquitination. Molecules and Cells.

[45] Ma J, Cai M, Mo Y (2021). The SPOP-ITCH signaling axis protects against prostate cancer metastasis. Frontiers in Oncology.

[46] Dixon KM, Lui GY, Kovacevic Z (2013). Dp44mT targets the AKT, TGF-beta and ERK pathways via the metastasis suppressor NDRG1 in normal prostate epithelial cells and prostate cancer cells. British Journal of Cancer.

[47] Gamell C, Bandilovska I, Gulati T (2019). E6AP promotes a metastatic phenotype in prostate cancer. iScience.

[48] Sundar R, Gudey SK, Heldin CH (2015). TRAF6 promotes TGFbeta-induced invasion and cell-cycle regulation via Lys63-linked polyubiquitination of Lys178 in TGFbeta type I receptor. Cell Cycle.

[49] Khusbu FY, Zhou X, Roy M (2020). Resveratrol induces depletion of TRAF6 and suppresses prostate cancer cell proliferation and migration. The International Journal of Biochemistry and Cell Biology.

[50] Singh R, Karri D, Shen H (2018). TRAF4-mediated ubiquitination of NGF receptor TrkA regulates prostate cancer metastasis[J]. The Journal of Clinical Investigation.

[51] Kwok WK, Ling MT, Lee TW (2005). Up-regulation of TWIST in prostate cancer and its implication as a therapeutic target. Cancer Research.

[52] Ruan D, He J, Li CF (2017). Skp2 deficiency restricts the progression and stem cell features of castration-resistant prostate cancer by destabilizing twist. Oncogene.

[53] Mickova A, Kharaishvili G, Kurfurstova D (2021). Skp2 and Slug are coexpressed in aggressive prostate cancer and inhibited by neddylation blockade. International Journal of Molecular Sciences.

[54] Mitsiades N (2013). A road map to comprehensive androgen receptor axis targeting for castration-resistant prostate cancer. Cancer Research.

[55] Chen CD, Welsbie DS, Tran C (2004). Molecular determinants of resistance to antiandrogen therapy. Nature Medicine.

[56] An J, Wang C, Deng Y (2014). Destruction of full-length androgen receptor by wild-type SPOP, but not prostate-cancer-associated mutants. Cell Reports.

[57] Geng C, Rajapakshe K, Shah SS (2014). Androgen receptor is the key transcriptional mediator of the tumor suppressor SPOP in prostate cancer. Cancer Research.

[58] Lin HK, Wang L, Hu YC (2002). Phosphorylation-dependent ubiquitylation and degradation of androgen receptor by Akt require Mdm2 E3 ligase. The EMBO Journal.

[59] Li B, Lu W, Yang Q (2014). Skp2 regulates androgen receptor through ubiquitin-mediated degradation independent of Akt/mTOR pathways in prostate cancer. Prostate.

[60] Vummidi Giridhar P, Williams K, Vonhandorf AP (2019). Constant degradation of the androgen receptor by MDM2 conserves prostate cancer stem cell integrity. Cancer Research.

[61] Moon SJ, Jeong BC, Kim HJ (2018). DBC1 promotes castration-resistant prostate cancer by positively regulating DNA binding and stability of AR-V7. Oncogene.

[62] Xu K, Shimelis H, Linn DE (2009). Regulation of androgen receptor transcriptional activity and specificity by RNF6-induced ubiquitination. Cancer Cell.

[63] Békés M, Langley DR, Crews CM (2022). PROTAC targeted protein degraders: The past is prologue. Nature Review Drug Discovery.

[64] O’brien CA, Pollett A, Gallinger S (2007). A human colon cancer cell capable of initiating tumour growth in immunodeficient mice. Nature.

[65] Peitzsch C, Tyutyunnykova A, Pantel K (2017). Cancer stem cells: The root of tumor recurrence and metastases. Seminars in Cancer Biology.

[66] Yoo YA, Vatapalli R, Lysy B (2019). The role of castration-resistant Bmi1+Sox2+ cells in driving recurrence in prostate cancer. Journal of the National Cancer Institute.

[67] Jiao M, Qi M, Zhang F (2019). CUL4B regulates cancer stem-like traits of prostate cancer cells by targeting BMI1 via miR200b/c. Prostate.

[68] Gawlik-Rzemieniewska N, Bednarek I (2016). The role of NANOG transcriptional factor in the development of malignant phenotype of cancer cells. Cancer Biological Therapy.

[69] Wang X, Jin J, Wan F (2019). AMPK promotes SPOP-Mediated NANOG degradation to regulate prostate cancer cell stemness. Developmental Cell.

[70] Zhang J, Chen M, Zhu Y (2019). SPOP promotes nanog destruction to suppress stem cell traits and prostate cancer progression. Developmental Cell.

[71] Hanahan D, Weinberg RA (2011). Hallmarks of cancer: The next generation. Cell.

[72] Jia D, Lu M, Jung KH (2019). Elucidating cancer metabolic plasticity by coupling gene regulation with metabolic pathways. Proceedings of the National Academy of Sciences USA.

[73] Warburg O (1956). On the origin of cancer cells. Science.

[74] Fan Y, Ou L, Fan J (2020). PLCepsilon regulates metabolism and metastasis signaling via HIF-1alpha/MEK/ERK pathway in prostate cancer. The Journal of Cellular Physiology.

[75] Deberardinis RJ, Chandel NS (2016). Fundamentals of cancer metabolism.. Science Advances.

[76] Agarwal E, Goldman AR, Tang HY (2021). A cancer ubiquitome landscape identifies metabolic reprogramming as target of Parkin tumor suppression. Science Advances.

[77] Vila IK, Yao Y, Kim G (2017). A UBE2O-AMPKalpha2 axis that promotes tumor initiation and progression offers opportunities for therapy. Cancer Cell.

[78] Gong Y, Zack TI, Morris LG (2014). Pan-cancer genetic analysis identifies PARK2 as a master regulator of G1/S cyclins. Nature Genetics.

[79] Icard P, Fournel L, Wu Z (2019). Interconnection between metabolism and cell cycle in cancer. Trends in Biochemical Sciences.

[80] Nishiyama T, Ladurner R, Schmitz J (2010). Sororin mediates sister chromatid cohesion by antagonizing Wapl. Cell.

[81] Rankin S, Ayad NG, Kirschner MW (2005). Sororin, a substrate of the anaphase-promoting complex, is required for sister chromatid cohesion in vertebrates. Molecular Cell.

[82] Luo Z, Wang J, Zhu Y (2021). SPOP promotes CDCA5 degradation to regulate prostate cancer progression via the AKT pathway. Neoplasia.

[83] Teixeira LK, Wang X, Li Y (2015). Cyclin E deregulation promotes loss of specific genomic regions. Current Biology.

[84] Ju LG, Zhu Y, Long QY (2019). SPOP suppresses prostate cancer through regulation of CYCLIN E1 stability. Cell Death and Differentiation.

[85] Gao P, Hao JL, Xie QW (2022). PELO facilitates PLK1-induced the ubiquitination and degradation of Smad4 and promotes the progression of prostate cancer. Oncogene.

[86] Ren D, Dai Y, Yang Q (2019). Wnt5a induces and maintains prostate cancer cells dormancy in bone. Journal of Experimental Medicine.

[87] Stankiewicz E, Mao X, Mangham DC (2017). Identification of FBXL4 as a metastasis associated gene in prostate cancer. Scientific Reports.

[88] Yanagisawa K, Konishi H, Arima C (2010). Novel metastasis-related gene CIM functions in the regulation of multiple cellular stress-response pathways. Cancer Research.

[89] Satija YK, Bhardwaj A, Das S (2013). A portrayal of E3 ubiquitin ligases and deubiquitylases in cancer. International Journal of Cancer.

[90] Senft D, Qi J, Ronai ZA (2018). Ubiquitin ligases in oncogenic transformation and cancer therapy. Nature Reviews Cancer.

[91] Sakamoto KM, Kim KB, Kumagai A (2001). Protacs: Chimeric molecules that target proteins to the Skp1-Cullin-F box complex for ubiquitination and degradation. Proceedings of the National Academy of Sciences USA.

[92] Toure M, Crews CM (2016). Small-molecule PROTACS: New approaches to protein degradation. Angewandte Chemie International Edition.

[93] Li X, Song Y (2020). Proteolysis-targeting chimera (PROTAC) for targeted protein degradation and cancer therapy. Journal of Hematology and Oncology.

[94] Winter GE, Buckley DL, Paulk J (2015). DRUG DEVELOPMENT. Phthalimide conjugation as a strategy for in vivo target protein degradation. Science.

[95] Snyder LB, Neklesa TK, Chen X (2021). Discovery of ARV-110, a first in class androgen receptor degrading PROTAC for the treatment of men with metastatic castration resistant prostate cancer. Cancer Research.

[96] Nguyen TT, Kim JW, Choi HI (2022). Development of an LC-MS/MS Method for ARV-110, a PROTAC molecule, and applications to pharmacokinetic studies. Molecules.

[97] Salami J, Alabi S, Willard RR (2018). Androgen receptor degradation by the proteolysis-targeting chimera ARCC-4 outperforms enzalutamide in cellular models of prostate cancer drug resistance. Communications Biology.

[98] Lee GT, Nagaya N, Desantis J (2021). Effects of MTX-23, a Novel PROTAC of androgen receptor splice Variant-7 and androgen receptor, on CRPC resistant to second-line antiandrogen therapy. Molecular Cancer Therapeutics.

[99] Han X, Zhao L, Xiang W (2019). Discovery of highly potent and efficient PROTAC degraders of Androgen Receptor (AR) by employing weak binding affinity VHL E3 ligase ligands. Journal of Medicinal Chemistry.

[100] Kregel S, Wang C, Han X (2020). Androgen receptor degraders overcome common resistance mechanisms developed during prostate cancer treatment. Neoplasia.

[101] Chen L, Han L, Mao S (2021). Discovery of A031 as effective proteolysis targeting chimera (PROTAC) androgen receptor (AR) degrader for the treatment of prostate cancer. European Journal of Medicinal Chemistry.

[102] Han X, Wang C, Qin C (2019). Discovery of ARD-69 as a highly potent Proteolysis Targeting Chimera (PROTAC) degrader of Androgen Receptor (AR) for the treatment of prostate cancer. Journal of Medcinal Chemistry.

[103] Xiang W, Zhao L, Han X (2021). Discovery of ARD-2585 as an exceptionally Potent and Orally Active PROTAC degrader of androgen receptor for the treatment of advanced prostate cancer. Journal of Medicinal Chemistry.

[104] Han X, Zhao L, Xiang W (2021). Strategies toward discovery of potent and orally bioavailable proteolysis targeting chimera degraders of androgen receptor for the treatment of prostate cancer. Journal of Medicinal Chemistry.

[105] Lin W, Luo J, Sun Y (2018). ASC-J9((R)) suppresses prostate cancer cell invasion via altering the sumoylation-phosphorylation of STAT3. Cancer Letters.

[106] Park SY, Park JW, Lee GW (2018). Inhibition of neddylation facilitates cell migration through enhanced phosphorylation of caveolin-1 in PC3 and U373MG cells. BMC Cancer.

[107] Chen Y, Pan C, Wang X (2021). Silencing of METTL3 effectively hinders invasion and metastasis of prostate cancer cells. Theranostics.

[108] Deng R, Guo Y, Li L (2021). BAP1 suppresses prostate cancer progression by deubiquitinating and stabilizing PTEN. Molecular Oncology.

[109] Lee JE, Park CM, Kim JH (2020). USP7 deubiquitinates and stabilizes EZH2 in prostate cancer cells. Genetics and Molecular Biology.

[110] Park JM, Lee JE, Park CM (2019). USP44 promotes the tumorigenesis of prostate cancer cells through EZH2 Protein stabilization. Molecular Cells.

[111] Kim B, Kim H, Jung S (2020). A CTGF-RUNX2-RANKL axis in breast and prostate cancer cells promotes tumor progression in bone. Journal of Bone and Mineral Research.

